# Multicenter phase II study of neoadjuvant FOLFOXIRI followed by concurrent chemoradiotherapy in Chinese patients with high-risk rectal cancer

**DOI:** 10.1093/oncolo/oyag162

**Published:** 2026-04-28

**Authors:** Rashid N Lui, Simon Chu, Dennis C K Ng, Leung Li, Carmen C M Cho, Esther H Y Hung, Kwan Hung Wong, Frankie K F Mo, Eric C H Wong, Connie W C Hui, Daisy C M Lam, Joyce Suen, Wei Kang, Wing-Ming Ho, Kaori Futaba, Sophie Sok Fei Hon, Kelvin Yan, Simon S M Ng, Brigette Buig Yue Ma

**Affiliations:** Department of Medicine and Therapeutics, Prince of Wales Hospital, The Chinese University of Hong Kong, Hong Kong SAR China; Department of Clinical Oncology, State Key Laboratory of Translational Oncology, Sir YK Pao Centre for Cancer, Hong Kong Cancer Institute, Prince of Wales Hospital, The Chinese University of Hong Kong, Hong Kong SAR China; Department of Surgery, Prince of Wales Hospital, The Chinese University of Hong Kong, Hong Kong SAR China; Department of Surgery, Prince of Wales Hospital, The Chinese University of Hong Kong, Hong Kong SAR China; Department of Clinical Oncology, State Key Laboratory of Translational Oncology, Sir YK Pao Centre for Cancer, Hong Kong Cancer Institute, Prince of Wales Hospital, The Chinese University of Hong Kong, Hong Kong SAR China; Department of Imaging and Interventional Radiology, Prince of Wales Hospital, The Chinese University of Hong Kong, Hong Kong SAR China; Department of Imaging and Interventional Radiology, Prince of Wales Hospital, The Chinese University of Hong Kong, Hong Kong SAR China; Department of Clinical Oncology, State Key Laboratory of Translational Oncology, Sir YK Pao Centre for Cancer, Hong Kong Cancer Institute, Prince of Wales Hospital, The Chinese University of Hong Kong, Hong Kong SAR China; Department of Clinical Oncology, State Key Laboratory of Translational Oncology, Sir YK Pao Centre for Cancer, Hong Kong Cancer Institute, Prince of Wales Hospital, The Chinese University of Hong Kong, Hong Kong SAR China; Department of Clinical Oncology, State Key Laboratory of Translational Oncology, Sir YK Pao Centre for Cancer, Hong Kong Cancer Institute, Prince of Wales Hospital, The Chinese University of Hong Kong, Hong Kong SAR China; Department of Clinical Oncology, State Key Laboratory of Translational Oncology, Sir YK Pao Centre for Cancer, Hong Kong Cancer Institute, Prince of Wales Hospital, The Chinese University of Hong Kong, Hong Kong SAR China; Department of Clinical Oncology, State Key Laboratory of Translational Oncology, Sir YK Pao Centre for Cancer, Hong Kong Cancer Institute, Prince of Wales Hospital, The Chinese University of Hong Kong, Hong Kong SAR China; Department of Clinical Oncology, State Key Laboratory of Translational Oncology, Sir YK Pao Centre for Cancer, Hong Kong Cancer Institute, Prince of Wales Hospital, The Chinese University of Hong Kong, Hong Kong SAR China; Department of Anatomical and Cellular Pathology, The Chinese University of Hong Kong, Hong Kong SAR China; Department of Clinical Oncology, State Key Laboratory of Translational Oncology, Sir YK Pao Centre for Cancer, Hong Kong Cancer Institute, Prince of Wales Hospital, The Chinese University of Hong Kong, Hong Kong SAR China; Department of Surgery, Prince of Wales Hospital, The Chinese University of Hong Kong, Hong Kong SAR China; Department of Surgery, Prince of Wales Hospital, The Chinese University of Hong Kong, Hong Kong SAR China; Department of Clinical Oncology, State Key Laboratory of Translational Oncology, Sir YK Pao Centre for Cancer, Hong Kong Cancer Institute, Prince of Wales Hospital, The Chinese University of Hong Kong, Hong Kong SAR China; Department of Surgery, Prince of Wales Hospital, The Chinese University of Hong Kong, Hong Kong SAR China; Department of Clinical Oncology, State Key Laboratory of Translational Oncology, Sir YK Pao Centre for Cancer, Hong Kong Cancer Institute, Prince of Wales Hospital, The Chinese University of Hong Kong, Hong Kong SAR China

**Keywords:** locally advanced rectal cancer, LARC, FOLFOXIRI, prognostic marker, IGF2

## Abstract

**Background:**

We investigated the feasibility of adding neoadjuvant FOLFOXIRI to chemoradiotherapy (CRT) in Chinese patients with high-risk, locally advanced rectal adenocarcinoma (LARC) and examined the prognostic significance of IGF2 and L1CAM expression.

**Methods:**

Eligible patients had non-metastatic, T3/T4 disease with or without nodal involvement, threatened circumferential resection margin (CRM) and/or sphincter involvement, received 4 cycles of a modified FOLFOXIRI regimen, followed by CRT, surgery, then adjuvant chemotherapy. Co-primary endpoints were objective response rate (ORR) and pathologic complete response rate (pCR). Secondary endpoints included overall survival (OS), relapse-free survival (RFS) and safety. Archival biopsies were analyzed for IGF2 and L1CAM expression using immunostaining.

**Results:**

Forty patients were enrolled with median age of 60 years and median follow up of 72.7 months. The ORR of FOLFOXIRI and CRT was 30.8% and 64.1%, respectively in 39 patients evaluated, 71.8% of them exhibiting TNM downstaging. For the entire cohort, the pCR rate was 20.5% and CRM was negative in 30 patients. The 3-year and 5-year OS were 79.5% and 59.5%, respectively. The 3-year RFS was 72.4%. Grade 3-4 toxicities to FOLFOXIRI were diarrhea (13%), neutropenia (8%), and vomiting (5%). Grade 3-4 toxicities to CRT were diarrhea (3%), rectal hemorrhage (3%), and sexual dysfunction (3%). High IGF2 expression was negatively correlated with disease-free survival (*P* = .0176) but not OS.

**Conclusions:**

Neoadjuvant modified FOLFOXIRI followed by concurrent capecitabine-RT and surgery was effective with manageable toxicities in Chinese patients with high risk LARC. Exploratory analyses showed that IGF2 expression may be a negative prognostic factor in LARC. (NCT01941641)

Lessons Learned

- Among Chinese patients with high-risk locally advanced rectal cancer (LARC), neoadjuvant FOLFOXIRI followed by capecitabine-based chemoradiotherapy yielded a pathologic complete response rate of 20.5%
- Toxicities were manageable with no treatment-related deaths or perioperative mortality encountered; overall, 24 out of 39 patients completed all planned treatment schedules
- IGF2 expression was associated with a detrimental impact on disease-free survival and may warrant further investigation as a negative prognostic factor for LARC

**Table:** 

**Trial information**	
**Disease**	High-risk, locally advanced rectal adenocarcinoma (LARC)
**Stage of Disease/Treatment**	I-III
**Prior therapy**	None
**Type of study**	Phase II
**Primary endpoints**	Co-primary endpoints were objective response rate (ORR) and pathologic complete response rate (pCR)
**Secondary endpoints**	Overall survival (OS), relapse-free survival (RFS), and safety
**Additional details of endpoints or study design:** Archival biopsies were analyzed for IGF2 and L1CAM expression using immunostaining	

**Table:** 

**Drug information**	
**Generic/Working name**	Modified FOLFOXIRI
**Company name**	Not applicable
**Drug type**	Chemotherapy
**Drug class**	5-flourouracil, platinum, DNA topoisomerase I inhibitor
**Dose**	Single arm: 4 cycles of a modified FOLFOXIRI regimen (Day, D1: CPT-11 165 mg/m^2^, D1: oxaliplatin 85 mg/m^2^, D1-2 200 mg/m^2^ leucovorin, D1-2 5FU 400 mg/m^2^ over 30 minutes, then 5Fluorauracil, 5FU 600 mg/m^2^ over 44 hours together with growth factor support, followed by capecitabine-RT (CRT, capecitabine 825 mg/m2 twice daily given concurrently with pre-operative RT of 50.4 Gy in 28 fractions over 5 weeks). Surgery was planned at 8-10 weeks after CRT, followed by adjuvant XELOX for 4 cycles.
**Route**	IV
**Schedule of administration**	Days 1-2 of a 14-day cycle

**Table:** 

Patient characteristics	Patients received FOLFOXIRI *N* = 39 (%)	
**Number of patients, male**		32 (82)
**Number of patients, female**		7 (18)
**Median age**		60 (43-78)
**Median follow-up time**		72.7 months (95% CI 62.6-78.2)
**Performance status**		
**ECOG 0**		23 (59)
**ECOG 1**		16 (41)
**T stage**		
**T2**		1 (2.6)
**T3**		26 (66.7)
**T4a**		4 (10.3)
**T4b**		8 (20.5)
**N stage**		
**N0**		4 (10.3)
**N1**		22 (56.4)
**N2**		13 (33.3)
**Overall stage**		
**Stage IIA**		2 (5.1)
**Stage IIC**		2 (5.1)
**Stage IIIB**		25 (64.1)
**Stage IIIC**		10 (25.7)
**Presence of EMVI**		
**Yes**		20 (51.3)
**No**		15 (39.5)
**Unknown**		4 (10.3)
**Reason for recommending neoadjuvant chemotherapy**		
**Threatened margin**		30 (76.9)
**Inoperable**		1 (2.6)
**Sphincter salvage**		2 (5.1)
**Threatened margin and sphincter**		6 (15.4)
**Tumor location**		
**Lower**		22 (56.4)
**Lower to mid**		3 (7.7)
**Mid**		8 (20.5)
**Mid to upper**		3 (7.7)
**Upper**		3 (7.7)

Low rectum: 0-7 cm from anal verge; mid rectum: 7-12 cm from anal verge; upper rectum: 12-15 cm from anal verge.

Abbreviations: ECOG, Eastern Cooperative Oncology Group; EMVI, extramural vascular invasion.

**Table:** 

Patient characteristics	Patients underwent surgery *N* = 31 (%)
**Name of surgery**	
**Anterior resection**	11 (35.5)
**Laparoscopic transanal TME**	9 (29)
**Abdominal perineal resection**	7 (22.6)
**Laparoscopic low anterior resection**	2 (6.5)
**Pelvic exenteration**	1 (3.2)
**Unknown surgery**	1 (3.2)
**Postoperative pathologic staging**	
**Stage 0**	8 (25.8)
**Stage I**	5 (16.1)
**Stage IIA**	7 (22.6)
**Stage IIC**	2 (6.5)
**Stage IIIA**	2 (6.5)
**Stage IIIB**	5 (16.1)
**Stage IIIC**	2 (6.5)

Abbreviation: TME, total mesorectal excision.

**Table:** 

**Primary assessment method**	
**Title**	Co-primary endpoints of objective response rate (ORR) and pathologic complete response rate (pCR)
**Number of patients enrolled**	40
**Number of patients evaluable for toxicity**	39
**Number of patients evaluable for efficacy**	39
**Evaluation method**	ORR per RECIST version 1.1 criteria; pCR as the absence of any detectable residual tumor cells in the resected specimen;
**Outcome notes**	

**Table:** 

**Adverse events**										
	All		G1		G2		G3		G4	
Adverse events	*N*	%	*N*	%	*N*	%	*N*	%	*N*	%
**During FOLFOXIRI**										
** Diarrhea**	28	72	18	46	5	13	5	13	0	0
** Nausea**	27	69	21	54	6	15	0	0	0	0
** Anorexia**	21	54	9	23	11	28	1	3	0	0
** Vomiting**	14	36	8	21	4	10	2	5	0	0
** Abdominal pain**	14	36	7	18	7	18	0	0	0	0
** Neutrophil count decreased**	12	31	0	0	9	23	2	5	1	3
** Fatigue**	11	28	9	23	2	5	0	0	0	0
** Peripheral sensory neuropathy**	11	28	10	26	1	3	0	0	0	0
** Alanine aminotransferase increased**	7	18	6	15	1	3	0	0	0	0
** Alopecia**	7	18	4	10	3	8	0	0	0	0
** Hypokalemia**	6	15	4	10	1	3	1	3	0	0
** Dizziness**	5	13	4	10	1	3	0	0	0	0
** General disorders and administration site conditions—Other**	5	13	5	13	0	0	0	0	0	0
** Injection site reaction**	5	13	3	8	2	5	0	0	0	0
** Weight loss**	5	13	5	13	0	0	0	0	0	0
** Constipation**	4	10	3	8	1	3	0	0	0	0
** Phlebitis**	4	10	2	5	2	5	0	0	0	0
** Platelet count decreased**	4	10	2	5	2	5	0	0	0	0
** Anemia**	3	8	3	8	0	0	0	0	0	0
** Gastrointestinal disorders—Other**	3	8	3	8	0	0	0	0	0	0
** Headache**	3	8	3	8	0	0	0	0	0	0
** Hyponatremia**	2	5	0	0	0	0	2	5	0	0
** Alkaline phosphatase increased**	2	5	1	3	1	3	0	0	0	0
** Allergic reaction**	2	5	2	5	0	0	0	0	0	0
** Dyspepsia**	2	5	1	3	1	3	0	0	0	0
** Fever**	2	5	2	5	0	0	0	0	0	0
** Hiccups**	2	5	2	5	0	0	0	0	0	0
** Hypoalbuminemia**	2	5	0	0	2	5	0	0	0	0
** Mucositis oral**	2	5	2	5	0	0	0	0	0	0
** Pruritus**	2	5	1	3	1	3	0	0	0	0
** Rash maculo-papular**	2	5	0	0	2	5	0	0	0	0
** Abdominal distension**	1	3	1	3	0	0	0	0	0	0
** Anal pain**	1	3	0	0	1	3	0	0	0	0
** Bone pain**	1	3	1	3	0	0	0	0	0	0
** Creatinine increased**	1	3	0	0	1	3	0	0	0	0
** Dry mouth**	1	3	1	3	0	0	0	0	0	0
** Dysgeusia**	1	3	1	3	0	0	0	0	0	0
** Edema limbs**	1	3	1	3	0	0	0	0	0	0
** Febrile neutropenia**	1	3	0	0	0	0	0	0	1	3
** Flushing**	1	3	1	3	0	0	0	0	0	0
** Gastroesophageal reflux disease**	1	3	1	3	0	0	0	0	0	0
** Insomnia**	1	3	0	0	1	3	0	0	0	0
** Metabolism and nutrition disorders—Other**	1	3	0	0	1	3	0	0	0	0
** Myalgia**	1	3	1	3	0	0	0	0	0	0
** Nervous system disorders—Other**	1	3	1	3	0	0	0	0	0	0
** Pain**	1	3	1	3	0	0	0	0	0	0
** Psychiatric disorders—Other**	1	3	1	3	0	0	0	0	0	0
** Rectal hemorrhage**	1	3	1	3	0	0	0	0	0	0
** Vertigo**	1	3	0	0	1	3	0	0	0	0
**During chemoradiotherapy**										
** Diarrhea**	24	65	17	46	6	16	1	3	0	0
** Dermatitis radiation**	22	59	13	35	9	24	0	0	0	0
** Renal and urinary disorders—Other**	20	54	19	51	1	3	0	0	0	0
** Anal pain**	12	32	7	19	5	14	0	0	0	0
** Urinary frequency**	11	30	10	27	1	3	0	0	0	0
** Anorexia**	10	27	8	22	2	5	0	0	0	0
** Abdominal pain**	9	24	9	24	0	0	0	0	0	0
** Gastrointestinal disorders—Other**	9	24	9	24	0	0	0	0	0	0
** Alopecia**	7	19	4	11	3	8	0	0	0	0
** Palmar-plantar erythrodysesthesia syndrome**	7	19	6	16	1	3	0	0	0	0
** Pruritus**	7	19	7	19	0	0	0	0	0	0
** Blood bilirubin increased**	5	14	5	14	0	0	0	0	0	0
** Hypoalbuminemia**	5	14	3	8	2	5	0	0	0	0
** Anemia**	4	11	2	5	2	5	0	0	0	0
** Fatigue**	4	11	4	11	0	0	0	0	0	0
** Peripheral sensory neuropathy**	4	11	4	11	0	0	0	0	0	0
** Alanine aminotransferase increased**	3	8	3	8	0	0	0	0	0	0
** Constipation**	3	8	3	8	0	0	0	0	0	0
** Mucositis oral**	3	8	3	8	0	0	0	0	0	0
** Neutrophil count decreased**	3	8	0	0	3	8	0	0	0	0
** Skin infection**	3	8	1	3	2	5	0	0	0	0
** Rectal hemorrhage**	2	5	1	3	0	0	1	3	0	0
** Alkaline phosphatase increased**	2	5	2	5	0	0	0	0	0	0
** Cystitis noninfective**	2	5	2	5	0	0	0	0	0	0
** Dry skin**	2	5	2	5	0	0	0	0	0	0
** General disorders and administration site conditions—Other**	2	5	2	5	0	0	0	0	0	0
** Hypokalemia**	2	5	1	3	1	3	0	0	0	0
** Nausea**	2	5	2	5	0	0	0	0	0	0
** Pelvic pain**	2	5	2	5	0	0	0	0	0	0
** Phlebitis**	2	5	2	5	0	0	0	0	0	0
** Platelet count decreased**	2	5	2	5	0	0	0	0	0	0
** Skin and subcutaneous tissue disorders—Other**	2	5	1	3	1	3	0	0	0	0
** Vomiting**	2	5	2	5	0	0	0	0	0	0
** Weight loss**	2	5	2	5	0	0	0	0	0	0
** Delayed orgasm**	1	3	0	0	0	0	1	3	0	0
** Anal hemorrhage**	1	3	1	3	0	0	0	0	0	0
** Bloating**	1	3	1	3	0	0	0	0	0	0
** Disseminated intravascular coagulation**	1	3	1	3	0	0	0	0	0	0
** Dysgeusia**	1	3	1	3	0	0	0	0	0	0
** Dyspepsia**	1	3	1	3	0	0	0	0	0	0
** Flatulence**	1	3	0	0	1	3	0	0	0	0
** Hemorrhoids**	1	3	1	3	0	0	0	0	0	0
** Injection site reaction**	1	3	1	3	0	0	0	0	0	0
** Insomnia**	1	3	0	0	1	3	0	0	0	0
** Lower gastrointestinal hemorrhage**	1	3	1	3	0	0	0	0	0	0
** Pain**	1	3	1	3	0	0	0	0	0	0
** Perineal pain**	1	3	1	3	0	0	0	0	0	0
** Skin hyperpigmentation**	1	3	1	3	0	0	0	0	0	0
** Urinary tract infection**	1	3	0	0	1	3	0	0	0	0
** Urinary tract pain**	1	3	1	3	0	0	0	0	0	0
**Postoperative period**										
** Pain**	9	31	4	14	4	14	1	3	0	0
** Nausea**	7	24	6	21	1	3	0	0	0	0
** Gastrointestinal disorders—Other**	4	14	1	3	2	7	1	3	0	0
** Abdominal distension**	4	14	1	3	3	10	0	0	0	0
** Abdominal pain**	4	14	1	3	3	10	0	0	0	0
** Peripheral sensory neuropathy**	4	14	3	10	1	3	0	0	0	0
** Anemia**	3	10	1	3	0	0	2	7	0	0
** Fever**	3	10	1	3	2	7	0	0	0	0
** Vomiting**	3	10	2	7	1	3	0	0	0	0
** Weight loss**	3	10	0	0	3	10	0	0	0	0
** Injury poisoning and procedural complications—Other**	2	7	0	0	1	3	1	3	0	0
** Insomnia**	2	7	0	0	2	7	0	0	0	0
** Anxiety**	1	3	0	0	0	0	1	3	0	0
** Hematoma**	1	3	0	0	0	0	1	3	0	0
** Hypertension**	1	3	0	0	0	0	1	3	0	0
** Urine output decreased**	1	3	0	0	0	0	1	3	0	0
** Wound infection**	1	3	0	0	0	0	1	3	0	0
** Alopecia**	1	3	1	3	0	0	0	0	0	0
** Anorexia**	1	3	1	3	0	0	0	0	0	0
** Back pain**	1	3	1	3	0	0	0	0	0	0
** Bloating**	1	3	0	0	1	3	0	0	0	0
** Cough**	1	3	1	3	0	0	0	0	0	0
** Dehydration**	1	3	0	0	1	3	0	0	0	0
** Diarrhea**	1	3	1	3	0	0	0	0	0	0
** Dizziness**	1	3	1	3	0	0	0	0	0	0
** Dyspnea**	1	3	0	0	1	3	0	0	0	0
** Fatigue**	1	3	1	3	0	0	0	0	0	0
** Flatulence**	1	3	0	0	1	3	0	0	0	0
** Gastric anastomotic leak**	1	3	1	3	0	0	0	0	0	0
** Hiccups**	1	3	0	0	1	3	0	0	0	0
** Hypoalbuminemia**	1	3	0	0	1	3	0	0	0	0
** Hypomagnesemia**	1	3	1	3	0	0	0	0	0	0
** Hypotension**	1	3	0	0	1	3	0	0	0	0
** Infections and infestations—Other**	1	3	0	0	1	3	0	0	0	0
** Rectal hemorrhage**	1	3	1	3	0	0	0	0	0	0
** Renal and urinary disorders—Other**	1	3	1	3	0	0	0	0	0	0
** Reproductive system and breast disorders—Other**	1	3	0	0	1	3	0	0	0	0
** Urinary frequency**	1	3	0	0	1	3	0	0	0	0
** Urinary retention**	1	3	1	3	0	0	0	0	0	0

## Introduction

Despite advances in neoadjuvant treatment (NAT) for locally advanced rectal cancer (LARC), the prognosis still remains suboptimal with a disease-free survival (DFS) rate of 70.6% at 3 years reported in a meta-analysis of randomized controlled trials.^1^ Recently, the UNICANCER-PRODIGE23 trial showed that a triplet chemotherapy regimen before preoperative chemoradiotherapy (CRT) improved overall survival (OS), and conferred sustained benefits in disease-free survival (DFS) and metastasis-free survival (MFS) when compared with preoperative CRT alone in LARC.^2^ This has led to increased utilization of total neoadjuvant therapy (TNT) approaches although substantial treatment variations still exist.^3^ In addition, there is a relative paucity of data from Asian patients.^4^ Thus, we performed this study to investigate the efficacy of adding neoadjuvant modified FOLFOXIRI before CRT in Chinese patients with high-risk LARC.

## Materials and methods

### Study design

This was a single-arm, prospective, phase II study involving 3 centers in Hong Kong–the Prince of Wales Hospital, North District Hospital and the Alice Ho Miu Ling Nethersole Hospital. All patients were managed by the for Lower Gastrointestinal Cancer New Territories East Cluster Multidisciplinary Team. Eligible patients had non-metastatic, T3/T4 disease with or without nodal involvement, threatened circumferential resection margin (CRM) and/or sphincter involvement on pre-treatment investigation which included magnetic resonance imaging (MRI) scan, endorectal ultrasound and contrast computed tomography. We hypothesized that neoadjuvant FOLFOXIRI followed by CRT in high-risk LARC improves objective response rates (ORR) and pathological complete response (pCR) rates.

The sample size estimation was performed as follows: from historical data reported by our group, the pCR rate was 13.8% with neoadjuvant chemoradiation.^5^ Assuming that pre-operative FOLFOXIRI would achieve a 25% pCR rate, in order to detect this difference using Fleming’s 1-stage phase II procedure, with 2-sided alpha level of 0.05 and 80% power, 33 patients are required. (further details in supplementary materials). Based on published studies, the overall response rate of FOLFOXIRI is estimated to be around 60%.^6^ Pre-operative FOLFOXIRI is assumed to be inactive if the overall response rate is at most 40%, and active if it is at least 60%. By Fleming’s 2-stage phase II procedure, with 2-sided alpha level of 0.05 and 80% power, a total of 35 patients are required. Allowing for dropouts, in total 40 patients are required.

The treatment schedule and endpoints can be found in the drug information table and trial information table, respectively. Based on a recent transcriptomic profiling study, we also conducted an exploratory analysis on the prognostic value of insulin-like growth factor 2 (IGF2) and L1 cell adhesion molecule (L1CAM)^7^ (methods in the supplementary materials).

### Statistical analysis

For continuous variables, median with range were provided and compared using Mann–Whitney *U* test between groups. For dichotomous variables, comparison between groups was performed using chi-square test or Fisher’s exact test. OS was defined as the date of study entry until death from any cause. DFS was defined as the date of study entry until disease recurrence or relapse or death from any cause. RFS was defined as the date of complete surgical resection until disease recurrence. The time-to-event data was estimated with the method of Kaplan–Meier analysis for survival data and compared using log-rank test. The hazard ratio (HR) with 95% CI was estimated with Cox Proportional hazard model. A 2-sided *P*-value < .05 was considered statistically significant. Statistical analysis was performed based on SAS version 9.4 (SAS Institute).

## Results

**Figure 1.**
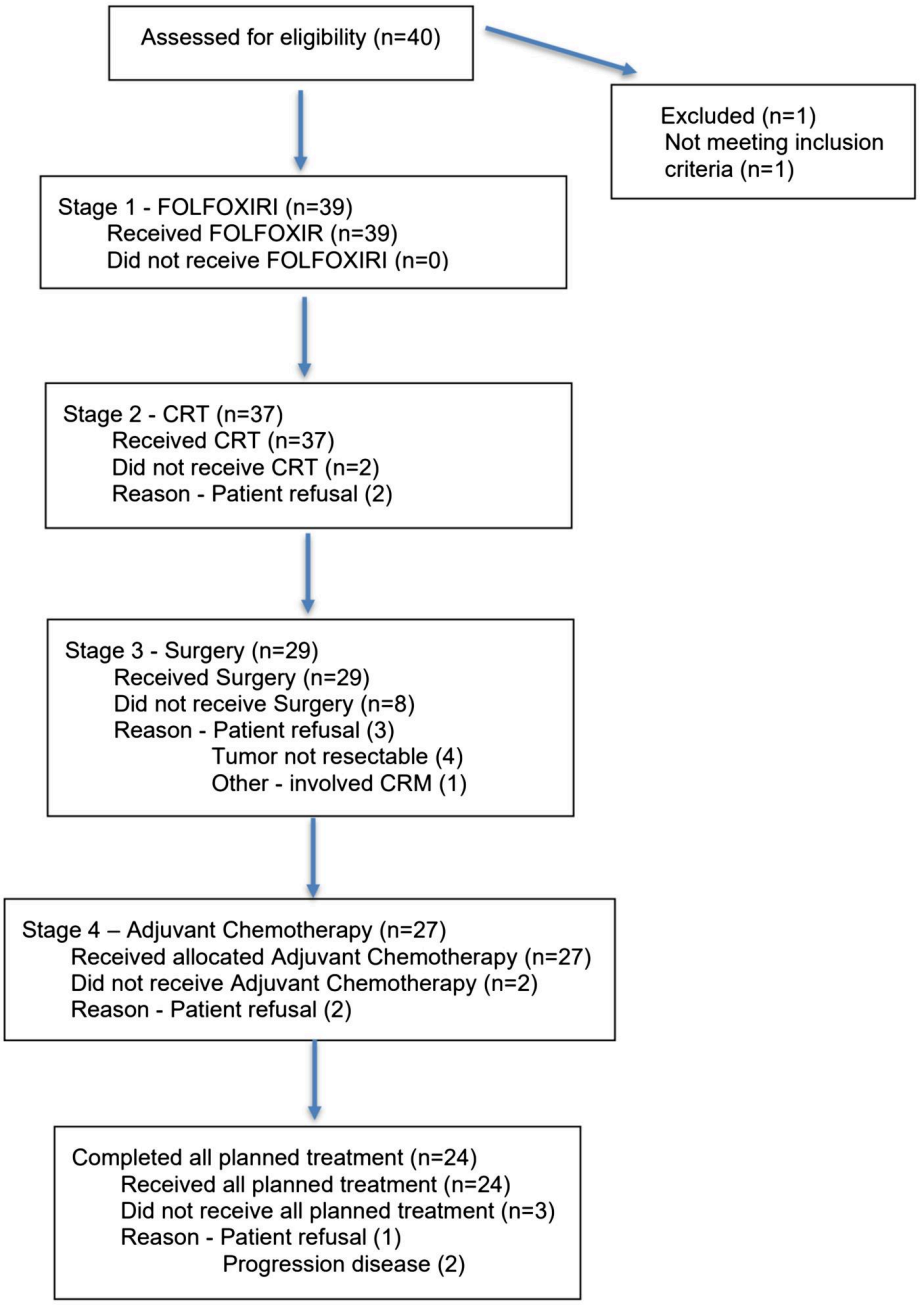
Patient flow diagram.

A total of 40 patients were enrolled from January 2014 to March 2019 with baseline characteristics found in the patient characteristics table. Patient treatment completion and drop out is illustrated in the flow diagram (Figure 1). One patient was excluded before starting treatment. Of the 39 patients evaluated for response, the ORR to neoadjuvant FOLFOXIRI was 30.8% (12 partial response [PR], 27 stable disease [SD]); ORR of CRT was 64.1% (25 PRs, 11 SDs, 1 progressive disease, 3 not assessed [NA], 1 not evaluable [NE]). MRI-based tumor regression grade showed improvement compared with baseline in 43.6% (17/39) of patients. Overall, 71.8% (28/39) had downstaging of disease, of whom 14 patients had downstaging of T stage, and 22 patients had downstaging of N stage. Among the eight patients who did not receive scheduled surgery, 2 patients were ultimately able to proceed after salvage chemotherapy. Of the 31 patients who underwent surgery, 21 patients required a stoma (6 were permanent). The type of surgery performed and overall stage post-operatively are also shown in the patient characteristics table.

**Figure 2.**
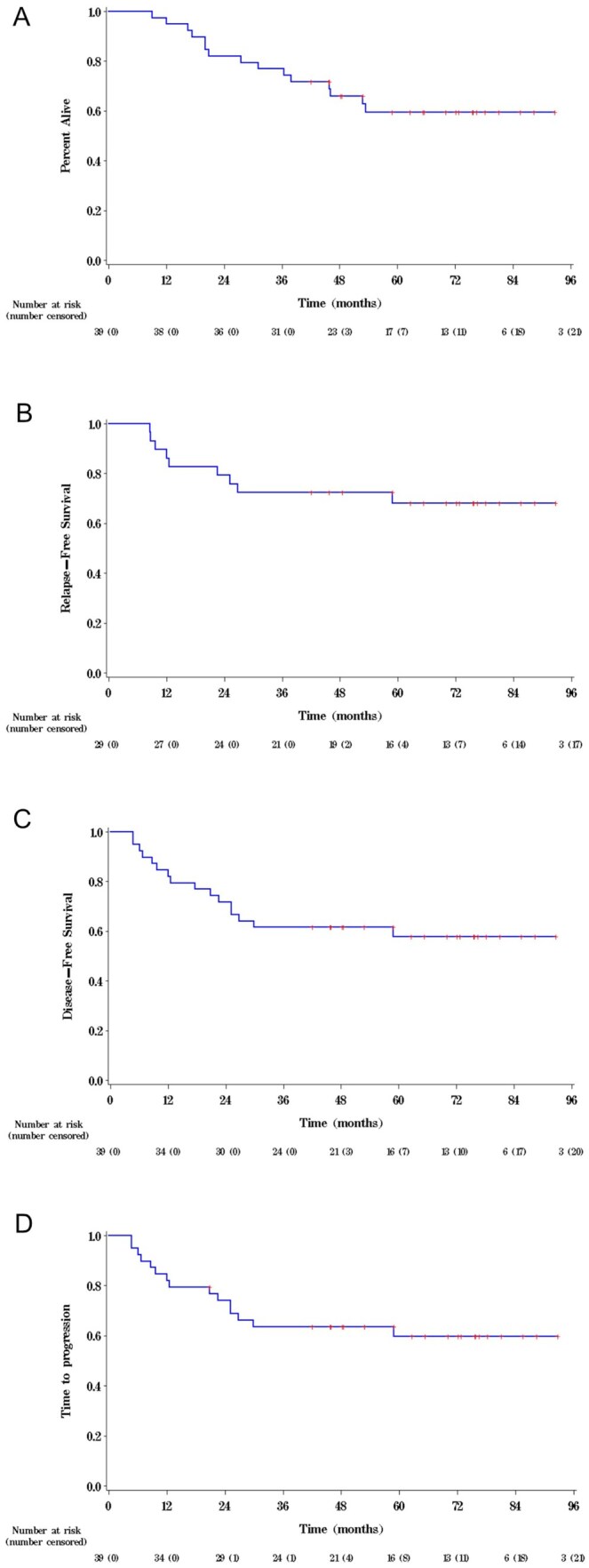
Kaplan–Meier curves for (A) overall survival, (B) recurrence-free survival, (C) disease-free survival, and (D) time to progression.

For patients who had evaluable pathology (post-surgery), the pCR rate was 25.8% (8/31), and the CRM was negative in 30 patients, whereas one patient had close margins. For the entire cohort the pCR rate was 20.5% (8/39). The median OS was not reached (NR) at the time of data cutoff in May 2023 (95% CI 46.0 months to NR) and the 1-year, 3-year, and 5-year OS were 97.4%, 79.5% and 59.5%, respectively (Figure 2A). The RFS was not reached (95% CI 59.4 months to NR) and the 1-year and 3-year RFS were 93.1% and 72.4%, respectively (Figure 2B). The median disease-free survival (DFS) was not reached (95% CI 26.8 to NR) and the 1-year and 3-year DFS were 87.2% and 61.5%, respectively (Figure 2C). The median time to local and distant failure was not reached (95% CI 29.8 to NR), the 1-year and 3-year rate of time to local or distant failure was 87.2% and 63.6%, respectively (Figure 2D) (only one patient was deemed to be a local failure).

Overall safety is presented in the adverse events table. Grade 3-4 toxicities were reported in 51.3% of patients (20/39). Two patients developed surgical complications within 30 days, namely bleeding and anastomotic leakage. No patients died within 30 days postoperatively.

## Discussion

Several landmark trials on TNT have already been published, such as CAO/ARO/AIO-12,^8^ OPRA^9^ and UNICANCER-PRODIGE23^2^ from the West, and the STELLAR^10^ and FOWARC^11^ trials from China. Our study demonstrates the safety and efficacy of systemic intensification with a modified FOLFOXIRI regimen in the neoadjuvant setting prior to CRT in Chinese patients. Being mindful of the limitations for cross trial comparisons, the pCR rate in this study for those who underwent surgery and for the entire cohort, was 25.8% and 20.5%, respectively, both of which are numerically superior to the reported 13.8% rate in a prior retrospective study utilizing CRT by our group.^5^ Our findings are numerically superior than the 17% pCR rate reported in the induction doublet chemotherapy arm of the CAO/ARO/AIO-12 trial^8^; similar to the 21.8% pCR rate reported in the short-course RT plus consolidation chemotherapy arm of the STELLAR trial^10^; but numerically inferior to the 28% pCR rate reported in the UNICANCER-PRODIGE23 trial. This difference may be attributable to the greater number (6) of modified FOLFIRINOX cycles that were used.^12^ We eagerly await the results of the Janus Rectal Cancer Trial, which will address the optimal strategy of consolidation chemotherapy intensification (doublet vs triplet) following long-course CRT.^13^

Another point of discussion is whether RT is even compulsory. Evidence from the FOWARC trial showed that mFOLFOX6 with or without radiation did not significantly improve 3-year DFS when compared with fluorouracil and RT in LARC.^11^ This was also shown in the more recent PROSPECT trial, where preoperative FOLFOX was non-inferior to preoperative CRT in terms of DFS for LARC patients eligible for sphincter-sparing surgery, with a similar pCR rate (21.9% vs 24.3%, respectively).^14^ However, real world evidence without RT has been inconsistent. A Singaporean study (majority of patients receiving doublet CAPOX) yielded a pCR rate of only 2.1%^15^; another study from China using FOLFOXIRI yielded a pCR rate of only 4.3%.^16^ These apparent differences are likely attributable to different study populations and highlight the importance of optimizing patient selection. Our study supports that RT is an essential component of TNT if achieving a respectable pCR is a treatment goal. Even so, one pitfall was our inability to detect CCR with existing diagnostic modalities after NAT which is a field for further research. Unfortunately, unless we can achieve the excellent responses of immunotherapy seen in mismatch repair deficient LARC,^17^ non-operative management with a watch-and-wait approach will unlikely be applicable for the majority of patients.^18^

The potential gain in therapeutic efficacy with systemic intensification must also be balanced against the increased toxicities of these regimens. Our regimen of 4 cycles of modified FOLFOXIRI was generally well tolerated in Chinese patients and is largely in keeping with the safety profile reported in UNICANCER-PRODIGE23.^2^ This, together with high treatment completion rates support the feasibility of using neoadjuvant FOLFOXIRI in carefully selected high-risk LARC patients. Of note, triplet regimens may be better tolerated in Asia due to ethnic differences in pharmacogenomics. Emerging evidence suggests deleterious variants of DPYD, one of the key genes responsible for the breakdown of fluoropyrimidines, is less common in East Asians.^19^

For the exploratory analyses, we were able to show the prognostic significance of IGF2, but not L1CAM in Chinese patients undergoing neoadjuvant FOLFOXIRI. However, neither biomarker was predictive of ORR to TNT.

Our study has several limitations. First, this is a non-randomized study with the possibility of inherent bias. Second, the sample size was relatively small. Third, this study was conducted between 2014 and 2019 prior to several contemporary studies looking at NAT in LARC. Fourth, our findings on IGF2 are exploratory and would require further validation. However, our results are promising and we believe that these findings warrant validation in a randomized setting for Asian populations.

## Conclusion

In summary, neoadjuvant modified FOLFOXIRI followed by concurrent chemoradiotherapy for high-risk LARC in Asian patients is a promising strategy showing good efficacy with acceptable toxicities. Larger-scale, randomized controlled trials will be needed for optimizing patient selection and to support wider adoption in the region. Tumoral expression of IGF2 warrants further investigation as a negative prognostic marker in this patient group.

## Supplementary Material

oyag162_Supplementary_Data

## Data Availability

Raw data that support the findings of this study are available from the corresponding author upon reasonable request.
