# Supplementary material for: Multicenter phase II study of neoadjuvant FOLFOXIRI followed by concurrent chemoradiotherapy in Chinese patients with high-risk rectal cancer
Source: Oncologist. 2026 Apr 28;31(6):oyag162. doi: 10.1093/oncolo/oyag162 (PMC13200789; doi:10.1093/oncolo/oyag162)

**Multicenter phase II study of Neoadjuvant FOLFOXIRI Followed by Concurrent Chemoradiotherapy in Chinese Patients with High-Risk Rectal Cancer**

**Authors**

Rashid N Lui1,2, Simon Chu3, Dennis CK Ng3, Leung Li2, Carmen CM Cho4, Esther HY Hung4, KH Wong2, Frankie KF Mo2, Eric CH Wong2, Connie WC Hui2, Daisy CM Lam2, Joyce Suen2, Wei Kang5, Wing-Ming Ho2, Kaori Futaba3, Sophie SF Hon3, Kelvin Yan2, Simon SM Ng3* and Brigette BY Ma2*‡ (*joint senior authors)

**Affiliations**

1. Department of Medicine and Therapeutics, Prince of Wales Hospital, The Chinese University of Hong Kong, Hong Kong SAR China

2. Department of Clinical Oncology, State Key Laboratory of Translational Oncology, Sir YK Pao Centre for Cancer, Hong Kong Cancer Institute, Prince of Wales Hospital, The Chinese University of Hong Kong, Hong Kong SAR China

3. Department of Surgery, Prince of Wales Hospital, The Chinese University of Hong Kong, Hong Kong SAR China

4. Department of Diagnostic and Interventional Radiology, Prince of Wales Hospital, The Chinese University of Hong Kong, Hong Kong SAR China

5. Department of Anatomical and Cellular Pathology, The Chinese University of Hong Kong, Hong Kong SAR China

*Corresponding author

Professor Brigette BY Ma

MBBS (Monash), MD (CUHK), FRACP, FHKCP, FHKAM (Medicine)

Department of Clinical Oncology, Hong Kong Cancer Institute, The Chinese University of Hong Kong

Hong Kong SAR, China

Tel: (852) 35052118

Email: [brigette@clo.cuhk.edu.hk](mailto:brigette@clo.cuhk.edu.hk)

‡Principal Investigator: Professor Brigette BY Ma

**Supplementary materials**

*Ethical statement*

The study was conducted in accordance with the Declaration of Helsinki and the International Standards of Good Clinical Practice with approval from the Joint CUHK-NTEC Institutional Review Board.

*Detailed calculation of sample size estimation*

The sample size calculation for pCR rate is as follows:

- Fleming’s 1-stage design

As mentioned previously, 33 patients will be enrolled. If the number of pCR in stage II is less than or equal to 6, then the study will be stopped and the alternative hypothesis rejected, if the number of responses in stage II is greater than or equal to 7, then the study will be stopped and the null hypothesis rejected.

- Fleming’s 2-stage design

Stage I: 20 patients will be enrolled, if the number of pCR in stage I is less than or equal to 2, then the study will be stopped and the alternative hypothesis rejected, if the number of pCR in stage I is greater than or equal to 6, then the study will be stopped and the null hypothesis rejected, if the number of pCR in stage I is greater than 2 and less than 6, then the study will be continued to stage II.

Stage II: An additional 13 patients will be enrolled. If the number of pCR in stage II is less than or equal to 6, then the study will be stopped and the alternative hypothesis rejected, if the number of responses in stage II is greater than or equal to 7, then the study will be stopped and the null hypothesis rejected.

*Immunostaining for biomarkers*

The expression of two biomarkers L1CAM and IGF2 was explored in pre-treatment archival biopsies. Tissue slides were sectioned from formalin-fixed paraffin embedded blocks for immunohistochemical (IHC) staining. For L1CAM staining, antigen retrieval was performed by 20 mins of 95oC of pH9 Tris buffer. For IGF2 staining, antigen retrieval was done by incubation with proteinase K for 15mins. The sections were then incubated with either 1:100 anti-L1CAM antibody (clone 14.10, Biolegend) or 1:100 anti-IGF2 antibody (clone S1F2, Millipore) at 4oC overnight and then incubated with DAKO REAL Envision HRP antibodies. The tissue staining was visualized in brown with DAB as a substrate followed by counterstaining with Mayer’s haematoxylin and then mounted by DPX Mountant. The staining intensity was blind scored by a senior staff member in our pathology department. For L1CAM, the scoring was based on the staining in cancer cells only. For IGF2, the scoring was based on the overall abundance in tumor tissues, including cancer and stromal cells. The staining intensity was scored as negative (0), weak (1), moderate (2) and strong (3). The expressions of L1CAM and IGF2 were then correlated with DFS and OS.

Pre-treatment archival biopsies of 32 patients were obtained for the immunostaining of prognostic markers. L1CAM and IGF2 were overexpressed in 78% and 46.9% of tumor samples, while high overexpression of L1CAM and IGF2 were found in 50% and 15.6% of samples, respectively (Table S1); representative IHC staining images were shown in Figure S1. As shown in Table S1, there were no significant correlations between these two markers and best response to neoadjuvant FOLFOXIRI and CRT. There was a statistically significant correlation between IGF2 expression (grouped into high versus low expression) and disease progression using Fisher’s Exact test (p = 0.0229), as well as with death and progression (Fisher’s Exact test, p = 0.0344). IGF2 expression was associated with worse DFS (hazard ratio, HR 4.477, 95% CI 1.299-15.437, p=0.017) but not OS (HR 3.294, 95% CI 0.843-12.877, p=0.087) (Figure S2). L1CAM expression was not associated with either DFS (HR 1.317, 95% CI 0.401-4.324, p=0.650) or OS (HR 1.054, 95% CI 0.305-3.647, p=0.934) (Figure S2).

Table S1. Immunohistochemistry (IHC) scores for L1CAM and IGF2, their correlation with best response to neoadjuvant FOLFOXIRI and chemoradiotherapy, associations of each marker with progression, death, and the composite endpoint of progression or death

| IHC Score (N = 32) | 0 | | 1 | | 2 | | 3 | |
| --- | --- | --- | --- | --- | --- | --- | --- | --- |
|  | N | % | N | % | N | % | N | % |
| L1CAM | 7 | 21.9 | 9 | 28.1 | 9 | 28.1 | 7 | 21.9 |
| IGF2 | 17 | 53.1 | 10 | 31.3 | 4 | 12.5 | 1 | 3.1 |
| Grouped IHC score (N = 32) | Low (0+1) | | | | High (2+3) | | | |
|  | N | | % | | N | | % | |
| L1CAM | 16 | | 50 | | 16 | | 50 | |
| IGF2 | 27 | | 84.4 | | 5 | | 15.6 | |
| Stage I – Neoadjuvant chemotherapy | | | | | | | | |
| L1CAM | Grouped IHC score | | | | | | | |
|  | Low (0+1) N (%) | | High (2+3) N (%) | | Total | | p-value | |
| Responder | 5 (41.7) | | 7 (58.3) | | 12 | | 0.4652 | |
| Non-responder | 11 (55.0) | | 9 (45.0) | | 20 | |  | |
| IGF2 | Grouped IHC score | | | | | | | |
|  | Low (0+1) N (%) | | High (2+3) N (%) | | Total | | p-value | |
| Responder | 9 (75.0) | | 3 (25.0) | | 12 | | 0.2076 | |
| Non-responder | 18 (90.0) | | 2 (10.0) | | 20 | |  | |
| Stage II – Concurrent Chemoradiation | | | | | | | | |
| L1CAM | Grouped IHC score | | | | | | | |
|  | Low (0+1) N (%) | | High (2+3) N (%) | | Total | | p-value | |
| Responder | 11 (50.0) | | 11 (50.0) | | 22 | | 1.0000 | |
| Non-responder | 5 (50.0) | | 5 (50.0) | | 10 | |  | |
| IGF2 | Grouped IHC score | | | | | | | |
|  | Low (0+1) N (%) | | High (2+3) N (%) | | Total | | p-value | |
| Responder | 19 (86.4) | | 3 (13.6) | | 22 | | 0.3441 | |
| Non-responder | 8 (80.0) | | 2 (20.0) | | 10 | |  | |
| Associations of markers with progression, death and composite endpoint of progression or death | | | | | | | | |
| L1CAM | Grouped IHC score | | | | | | | |
|  | Low (0+1) N (%) | | High (2+3) N (%) | | Total | | p-value | |
| Progressors | 4 (40.7) | | 6 (60.0) | | 12 | | 0.4456 | |
| Non-Progressors | 12 (54.5) | | 10 (45.5) | | 20 | |  | |
| Death | 5 (50.0) | | 5 (50.0) | | 10 | | 1.0000 | |
| Alive | 11 (50.0) | | 11 (50.0) | | 22 | |  | |
| Progression / Death | 5 (45.5) | | 6 (55.5) | | 11 | | 0.7097 | |
| Alive without disease | 11 (52.4) | | 10 (47.6) | | 21 | |  | |
| IGF2 | Grouped IHC score | | | | | | | |
|  | Low (0+1) N (%) | | High (2+3) N (%) | | Total | | p-value | |
| Progressors | 6 (60.0) | | 4 (40.0) | | 10 | | **0.0229** | |
| Non-Progressors | 21 (95.5) | | 1 (4.5) | | 22 | |  | |
| Death | 7 (70.0) | | 3 (30.0) | | 10 | | 0.1377 | |
| Alive | 20 (90.1) | | 2 (9.1) | | 22 | |  | |
| Progression or Death | 7 (63.6) | | 4 (36.4) | | 11 | | **0.0344** | |
| Alive without disease | 20 (95.2) | | 1 (4.8) | | 21 | |  | |

^p-value obtained by chi-square or fisher exact test^

Figure S1. Representative IHC staining images for L1CAM and IGF2 expression (scale bar = 200 micrometre).


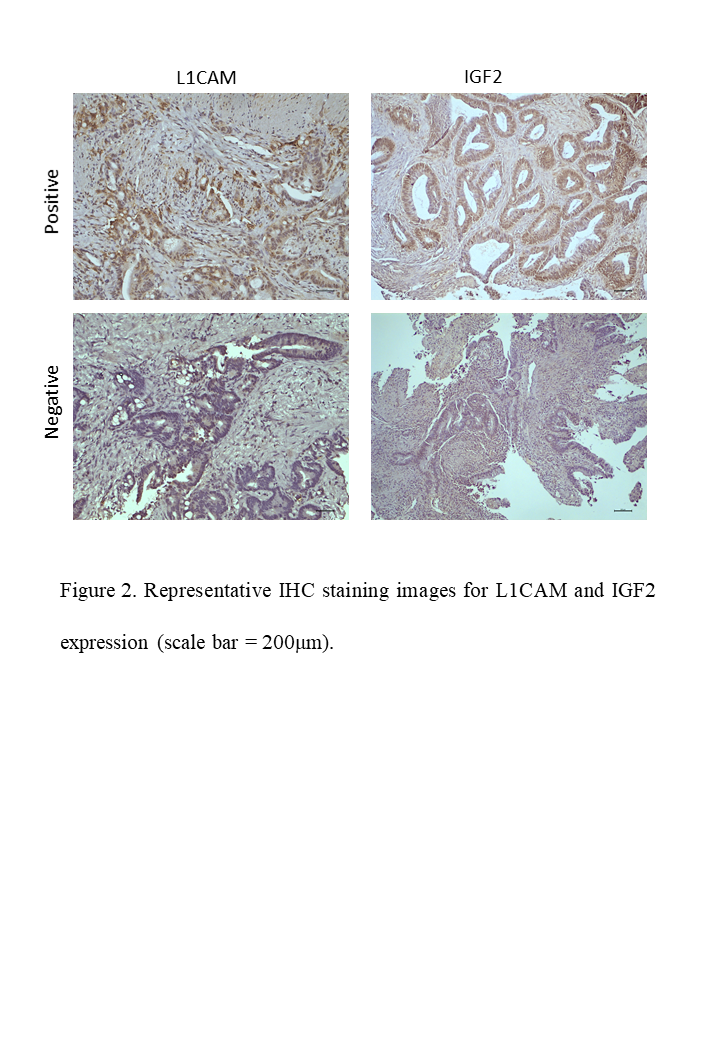


Figure S2. Associations of L1CAM and IGF2 expression with disease-free survival (DFS) and overall survival (OS): (A) DFS vs L1CAM, (B) DFS vs IGF2, (C) OS vs L1CAM, and (D) OS vs IGF2.


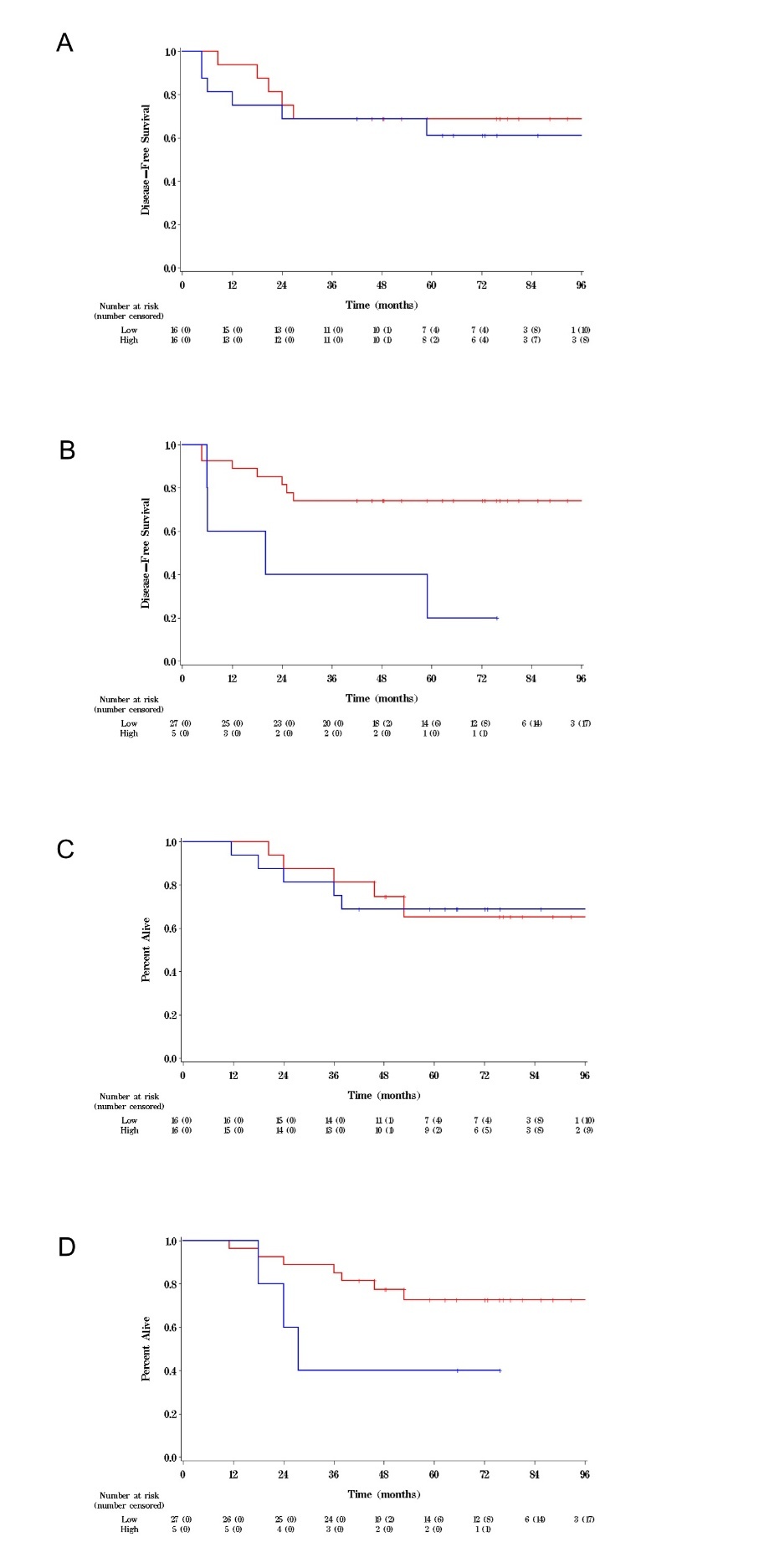

Supplement: oyag162_Supplementary_Data [file oyag162_supplementary_data.docx]
